# Epidemiology, antimicrobial resistance transmission, and One Health–based prevention of non-typhoidal *Salmonella* and *Campylobacter* infections: an updated literature review

**DOI:** 10.3389/fmicb.2026.1809070

**Published:** 2026-03-27

**Authors:** Guiliang Zheng, Lingling Chen, Guanghui Dong, Kun Ye, Zhe Liu, Lei Wang

**Affiliations:** 1 College of Resources and Environment, Shandong Agricultural University, Taian, Shandong, China; 2 School of Basic Medical Sciences, Tianjin Medical University, Tianjin, China; 3 Department of Occupational and Environmental Health, School of Public Health, Sun Yat-sen University, Guangzhou, China; 4 Department of Nephrology, Guangxi Clinical Research Center for Chronic Kidney Disease, People's Hospital of Guangxi Zhuang Autonomous Region, Nanning, Guangxi, China; 5 Geological Survey of Guangxi Zhuang Autonomous Region, Nanning, China; 6 Medical Geological Engineering Center of Guangxi Zhuang Autonomous Region, Nanning, China

**Keywords:** antimicrobial resistance, *Campylobacter* spp., foodborne disease, non-typhoidal *Salmonella*, One Health

## Abstract

Non-typhoidal *Salmonella* (NTS) and thermotolerant *Campylobacter* remain leading causes of bacterial gastroenteritis worldwide, with a disproportionate burden in low- and middle-income regions. This updated review synthesizes current evidence on their epidemiology, antimicrobial resistance (AMR) dynamics, and the role of genomic epidemiology within a One Health framework. The global expansion of multidrug-resistant clones, driven largely by antimicrobial use in agriculture, increasingly compromises therapeutic options. Advances in whole-genome sequencing (WGS) have enhanced outbreak detection, source attribution, and tracking of AMR gene transmission across human, animal, and environmental reservoirs. Recent large-scale genomic analyses further clarify the temporal evolution and international dissemination of high-risk lineages. We argue that effective mitigation requires coordinated genomic surveillance, strengthened antimicrobial stewardship, and targeted interventions across the food chain. Implementing genomics-informed, cross-sectoral strategies under the One Health paradigm is essential to reduce disease burden and contain the spread of resistance.

## 1 Introduction

The global burden of foodborne disease remains a persistent and formidable challenge to public health systems, food security, and economic development. Among the diverse array of biological hazards, bacterial pathogens are responsible for a significant proportion of illnesses, hospitalizations, and deaths. Non-typhoidal *Salmonella enterica* (NTS) and thermotolerant *Campylobacter* species, primarily *Campylobacter jejuni* and *Campylobacter coli*, stand out as preeminent causes of bacterial zoonotic gastroenteritis worldwide (Havelaar et al., 2015; Kirk et al., 2015). The World Health Organization (WHO) estimates that in 2010, these two pathogen groups were responsible for approximately 230 million illnesses and over 65,000 deaths globally, with the highest morbidity and mortality rates observed in Africa and Asia, highlighting stark regional disparities (Majowicz et al., 2010; World Health Organization, 2015). Updated EFSA and ECDC burden assessments and recent regional surveillance reports continue to indicate that non-typhoidal *Salmonella* and *Campylobacter* remain among the leading causes of bacterial foodborne disease globally (EFSA and ECDC, 2023). The clinical presentation typically involves acute, self-limiting gastroenteritis characterized by diarrhea, abdominal cramps, fever, and vomiting. However, these infections can also lead to severe invasive disease, particularly in vulnerable populations such as young children, the elderly, and the immunocompromised. Furthermore, a significant subset of patients experiences debilitating post-infectious sequelae, including reactive arthritis (ReA), irritable bowel syndrome (IBS), and Guillain-Barré syndrome (GBS) a serious neurological disorder strongly associated with preceding *C. jejuni* infection (Esan et al., 2017; Keithlin et al., 2015). These long-term consequences contribute substantially to the overall disability-adjusted life year (DALY) burden, underscoring that the impact of these pathogens extends far beyond the acute phase of illness.

The ecology and transmission dynamics of NTS and *Campylobacter* are deeply embedded within complex, interconnected systems encompassing animal agriculture, food processing, environmental pathways, and human consumption patterns. Their persistence and evolution are continuously shaped by a confluence of powerful drivers: intensification of livestock production, globalized food trade networks, climate change, and shifting consumer preferences (Hellberg and Chu, 2016; Jones et al., 2013). These factors not only facilitate the spread of established (“enduring”) pathogen strains but also create niches for the emergence of novel variants or the recognition of previously overlooked hazards (Mather et al., 2024). Compounding this biological complexity is the escalating crisis of antimicrobial resistance (AMR). The widespread and often indiscriminate use of antimicrobial agents in veterinary medicine for therapy, prophylaxis, and growth promotion, coupled with their use in human medicine, exerts immense selective pressure. This has led to the rapid emergence and global dissemination of multidrug-resistant (MDR) clones of NTS and *Campylobacter*, critically compromising the efficacy of first- and second-line therapeutic agents and posing a dire threat to modern medicine (CDC, 2019; Van Boeckel et al., 2015).

Addressing this multifaceted challenge necessitates a fundamental paradigm shift from isolated, sector-specific interventions to a holistic and integrated approach. The One Health concept, which recognizes the intrinsic interconnectedness of human, animal, and environmental health, provides the essential framework for such a strategy (Adisasmito et al., 2022). Central to operationalizing this framework in the modern era is the application of advanced genomic tools. The advent of affordable, high-throughput whole-genome sequencing (WGS) and metagenomics has transformed microbiological surveillance from a phenotypic descriptive exercise into a powerful, predictive science (Deng et al., 2016; Jackson et al., 2016). These technologies offer unparalleled resolution for outbreak investigation, source attribution, tracking the flow of resistance genes across ecosystems, and identifying emerging threats with precision.

This review aims to provide a comprehensive and updated synthesis of the current state of knowledge regarding NTS and *Campylobacter* infections. It will meticulously examine their global and regional epidemiology, with particular attention to data from under-represented regions such as the Arab world, as illustrated in recent descriptive reviews (Habib et al., 2021). It will delve deeply into the mechanisms, trends, and drivers of AMR, informed by groundbreaking large-scale genomic analyses that are mapping the global evolution of resistance (Jia et al., 2025; Wang et al., 2025). The review will critically evaluate the revolutionary impact of genomic epidemiology on our understanding of transmission pathways and its vital role in shaping targeted interventions. Finally, it will consolidate evidence on prevention and control strategies, arguing persuasively for their foundation within a robust, proactive, and genomics-informed One Health framework. The ultimate goal is to outline a cohesive path forward for mitigating the enduring and evolving threat posed by these significant foodborne pathogens.

### 1.1 Methodology

This study conducted a comprehensive literature review on the epidemiology, antimicrobial resistance (AMR), genomic surveillance, and One Health–based control strategies of non-typhoidal *Salmonella* (NTS) and *Campylobacter*. Relevant studies published between January 2010 and March 2025 were systematically searched in the PubMed, Web of Science, and Scopus databases. The retrieved literature was screened and analyzed to summarize current research progress and identify key knowledge gaps related to the transmission, resistance patterns, and integrated prevention strategies of these pathogens.

## 2 Global and regional epidemiology of NTS and *Campylobacter*

### 2.1 Prevalence in animal reservoirs and the food chain

NTS and *Campylobacter* are primarily zoonotic pathogens with a broad range of animal hosts. Domesticated food animals, especially poultry (broilers and layers), constitute the most significant reservoirs for human infection. Pigs, cattle, and small ruminants also serve as important reservoirs for specific serovars or species. The pathogens typically colonize the gastrointestinal tract of these animals asymptomatically, leading to contamination of carcasses during slaughter and processing. This contamination can subsequently persist or even amplify at various stages of the food chain, including during cutting, packaging, distribution, and retail.

Global prevalence estimates in food animals and products vary considerably but consistently highlight poultry as a key vehicle. In the European Union, for instance, *Campylobacter* is frequently detected in broiler meat, with a high proportion of samples often exceeding contamination limits set for public health protection (EFSA and ECDC, 2023). Similarly, NTS, particularly serovars like Enteritidis and Typhimurium, are routinely identified in poultry flocks and products worldwide. Among different regions, the Arab world represents a particularly important case study due to historically underrepresented surveillance data, specific regional climatic challenges, and a high rate of poultry consumption, all of which may uniquely influence the One Health transmission cycle. In this context, a focused descriptive review of Arab countries-where surveillance systems are often fragmented-confirmed the widespread presence of these pathogens across the region (Habib et al., 2021). The review compiled studies showing NTS prevalence ranging from 1 to 60% in chicken meat samples across different Arab nations, with notable findings such as 16% prevalence in imported frozen chicken carcasses in Iraq and 60% in chicken parts in Egypt (Habib et al., 2021). For *Campylobacter*, reported prevalence in poultry products reached up to 75% in frozen chicken meat in Iraq and 71.4% in broiler samples in Morocco (Habib et al., 2021). These figures, though variable due to differences in methodology and sampling frames, unequivocally demonstrate endemic contamination within regional food supplies, mirroring a global pattern.

Beyond poultry, other food matrices are also implicated. Raw milk and dairy products made from unpasteurized milk are recognized sources of both pathogens. Fresh produce (fruits and vegetables) can become contaminated through contact with contaminated water, soil, or animal manure, leading to outbreaks. The complexity of modern, globalized food supply chains means that a contaminated ingredient sourced from one continent can lead to human illnesses on another, complicating epidemiological investigations and underscoring the need for international cooperation.

### 2.2 Burden of human disease: incidence and geographical disparities

The incidence of human campylobacteriosis and salmonellosis is heavily influenced by geographical, socioeconomic, and infrastructural factors. According to WHO estimates, the African and South-East Asian regions suffer the highest burden of foodborne disease per capita, including that caused by NTS and *Campylobacter* (World Health Organization, 2015). The Middle East and North Africa (MENA) region follows closely, reporting the third-highest estimated burden (Al Khatib and Kabir, 2025; Habib et al., 2021). In these regions, contributing factors likely include warmer climates, challenges in maintaining consistent cold chains, limited access to clean water, variable food safety regulations, and under-resourced public health surveillance systems. A substantial number of cases are sporadic and go undiagnosed or unreported, making true incidence difficult to ascertain.

In contrast, high-income countries in North America, Europe, and the Western Pacific have established robust, active surveillance networks that provide more accurate data, albeit still subject to under-ascertainment. Here, *Campylobacter* spp. have consistently been the most commonly reported gastrointestinal bacterial pathogen for over a decade. In the European Union, reported confirmed cases of campylobacteriosis far exceed those of salmonellosis (EFSA and ECDC, 2023). In the United States, data from the Foodborne Diseases Active Surveillance Network (FoodNet) consistently show *Campylobacter* and *Salmonella* as leading causes of foodborne illness, hospitalizations, and deaths (Scallan and Mahon, 2012). The disparity in reported rates between high- and low-income countries reflects differences in diagnostic capacity, healthcare access, and reporting efficiency as much as true differences in exposure risk. It highlights the “tip of the iceberg” phenomenon in global foodborne disease epidemiology.

### 2.3 Post-infectious sequelae: extending the disease burden

The public health impact of NTS and *Campylobacter* extends far beyond the acute episode of gastroenteritis. Post-infectious sequelae contribute significantly to the long-term DALY burden and represent a major concern for patients and healthcare systems. Reactive arthritis (ReA), an inflammatory joint condition, is a well-characterized sequela triggered by enteric infections, including those caused by NTS and *Campylobacter*. The incidence reported in studies varies dramatically, from less than 1% to over 60%, influenced by host genetics (notably HLA-B27 status), pathogen characteristics, and study design such as follow-up duration, case definitions. Irritable bowel syndrome (IBS) is another common outcome, with meta-analyses suggesting that approximately 10% of patients with bacterial gastroenteritis may develop new-onset IBS, a condition characterized by chronic abdominal pain and altered bowel habits that can persist for years (Esan et al., 2017; Thabane et al., 2010).

The most severe sequela is Guillain-Barré syndrome (GBS), an acute autoimmune polyradiculoneuropathy leading to ascending paralysis. The *C. jejuni* infection is the single most identifiable precipitating factor, implicated in 20–40% of GBS cases in many countries (Poropatich et al., 2010). The molecular mimicry between gangliosides in human nerve tissue and specific lipooligosaccharide structures on the surface of certain *C. jejuni* strains drives this autoimmune response. Although GBS occurs in only a small fraction (estimated 0.1–0.3‰) of *Campylobacter* infections, its severity and potential for permanent disability make it a critical public health concern. The factors that determine which patients develop these sequelae, beyond known associations like HLA-B27 for ReA or specific bacterial sialylation for GBS, remain an active area of research. A systematic review by Esan et al. found limited and inconsistent evidence regarding the role of antibiotic use or proton-pump inhibitors in sequela development, highlighting a significant knowledge gap that warrants further investigation (Esan et al., 2017).

## 3 Antimicrobial resistance: trends, drivers, and genomic insights

### 3.1 The escalating AMR crisis in zoonotic pathogens

Antimicrobial resistance in NTS and *Campylobacter* is not an emerging threat but an intensifying crisis. Resistance compromises the treatment of severe or invasive infections, leading to prolonged illness, increased risk of bloodstream invasion, higher hospitalization rates, treatment failure, and mortality. The primary driver of this resistance is the selective pressure exerted by the use of antimicrobial agents. In food animal production, antimicrobials are used for three main purposes: therapy (treating sick animals), prophylaxis (preventing disease in herds), and, in many countries still, growth promotion. The latter two categories, which often involve prolonged, low-dose administration, are particularly potent drivers for the selection and enrichment of resistant bacteria within animal reservoirs (McEwen and Collignon, 2018; Van Boeckel et al., 2015).

The transmission of resistant bacteria from animals to humans occurs predominantly through the consumption of contaminated food, direct contact with animals, or through environmental pathways contaminated with animal waste. Once established in the human population, resistant strains can spread from person to person. Of particular concern is resistance to antibiotics classified as critically or highly important for human medicine by the WHO, such as fluoroquinolones (ciprofloxacin), third-generation cephalosporins (ceftriaxone), and macrolides (azithromycin, erythromycin).

### 3.2 Regional AMR patterns and surveillance challenges

Regional surveillance data, while heterogeneous, reveals alarming trends. In the Arab world, studies compiled by Habib et al. indicate high levels of resistance among isolates from food and animals (Habib et al., 2021). For NTS, examples include *S. enteritidis* from chickens in Saudi Arabia showing high resistance to streptomycin, chloramphenicol, and sulfamethoxazole (Habib et al., 2021), and isolates in Egypt exhibiting high rates of resistance to nalidixic acid (a fluoroquinolone precursor) and tetracycline. For *Campylobacter* spp., high resistance to erythromycin and tetracycline has been reported in Tunisia and Morocco, and remarkably high ciprofloxacin resistance (85.4%) was noted in clinical *C. jejuni* isolates from the United Arab Emirates—among the highest reported globally at the time (Habib et al., 2021; Sonnevend et al., 2006).

Similar patterns are observed worldwide. In Asia and parts of Europe, high rates of fluoroquinolone resistance in *Campylobacter* from poultry and humans are common. In the US, resistance to ciprofloxacin in *Campylobacter* remains low in domestic isolates but is higher in isolates linked to international travel. For NTS, MDR serovars like *S. typhimurium* DT104 (historically) and more recently, monophasic *S. typhimurium* ST34, have achieved global distribution (Petrovska et al., 2016), carrying resistance genes to multiple drug classes. A critical challenge is the lack of standardized, comprehensive AMR surveillance in food animals and retail meat in many parts of the world, which hinders a complete global assessment and the evaluation of intervention impacts.

### 3.3 Global genomic atlases: unveiling the dynamics of resistance

Recent landmark studies leveraging large-scale genomic datasets have provided unprecedented insights into the global dynamics of AMR in these pathogens. Wang et al. (2025) constructed a comprehensive global atlas of AMR in *Salmonella* spanning over a century (1900–2023) by analyzing nearly 80,000 publicly available genomes (Wang et al., 2025). Using high-resolution approaches including core-genome multilocus sequence typing (cgMLST) and whole-genome single nucleotide polymorphism (wgSNP) analysis, the study reconstructed phylogenetic relationships, temporal emergence patterns, and intercontinental dissemination routes of multidrug-resistant (MDR) lineages.

Importantly, this genomic atlas identified key temporal shifts in resistance associated with historical changes in antimicrobial usage practices in both human medicine and livestock production. The expansion of globally disseminated MDR clones—including epidemic lineages such as monophasic *S. typhimurium* ST34 and fluoroquinolone-resistant S. Kentucky ST198—was shown to coincide with increased agricultural antimicrobial consumption. Furthermore, the study highlighted the role of mobile genetic elements, particularly IncHI2 and IncI1 plasmids, in mediating horizontal transfer of extended-spectrum β-lactamase (ESBL) genes across continents. These findings provide direct genomic evidence linking antimicrobial usage policies with evolutionary selection at a global scale.

Their prior work focusing on China further elucidated the temporal dynamics of predominant serovars and associated AMR profiles, demonstrating clonal expansion of resistant serovars such as *S. Indiana* and *S. enteritidis* within food animal production systems (Wang et al., 2023b). Together, these analyses illustrate how national-level surveillance feeds into global evolutionary narratives.

A parallel longitudinal genomic investigation by Jia et al. (2025) charted AMR trends in *Campylobacter* from 1954 to 2023 using large-scale genome collections (Jia et al., 2025). Through phylogenomic reconstruction and resistance determinant mapping, the study delineated evolutionary trajectories of resistance to fluoroquinolones and macrolides. Specifically, target-site mutations in DNA gyrase (gyrA T86I substitution) were identified as a dominant mechanism underlying fluoroquinolone resistance, while acquisition of 23S rRNA mutations and erm(B)-associated methylation contributed to macrolide resistance dissemination. These resistance determinants were shown to emerge repeatedly in geographically distinct regions, reflecting strong convergent selection under antimicrobial pressure.

Collectively, these genomic atlases demonstrate that AMR evolution is neither linear nor localized, but shaped by dynamic interactions between clonal expansion, horizontal gene transfer, and ecological fitness advantages of “high-risk” lineages. From a public health perspective, such datasets enable predictive surveillance—identifying emerging MDR clones before they become globally dominant—and support risk-based antimicrobial stewardship strategies across sectors.

### 3.4 The One Health cycle of resistance

Genomic epidemiology has been instrumental in elucidating the One Health cycle of AMR transmission. WGS allows researchers to trace not just the relatedness of bacterial strains but also the movement of specific resistance genes and their genetic contexts such as plasmids and integrons, across different reservoirs. Resistant bacteria selected in animal guts can contaminate meat at slaughter. From there, they can cause human infection, and the resistance genes can potentially be transferred to other bacteria in the human gut microbiome. Furthermore, animal waste (manure) used as fertilizer introduces resistant bacteria and genes into agricultural soils and water systems, creating environmental reservoirs. These genes can be taken up by environmental bacteria or re-enter the food chain via contaminated irrigation water for crops (He et al., 2020; Zhu et al., 2013).

This complex web underscores that interventions focused solely on human medicine are doomed to fail. Controlling AMR in zoonotic pathogens requires breaking the cycle at multiple points, primarily through responsible antimicrobial use in animal husbandry, effective waste management, and continued surveillance across all sectors.

## 4 Transmission dynamics of foodborne pathogens in the global food chain

### 4.1 Transmission pathways and diffusion mechanisms of foodborne pathogens in the food chain

**Figure 1:**
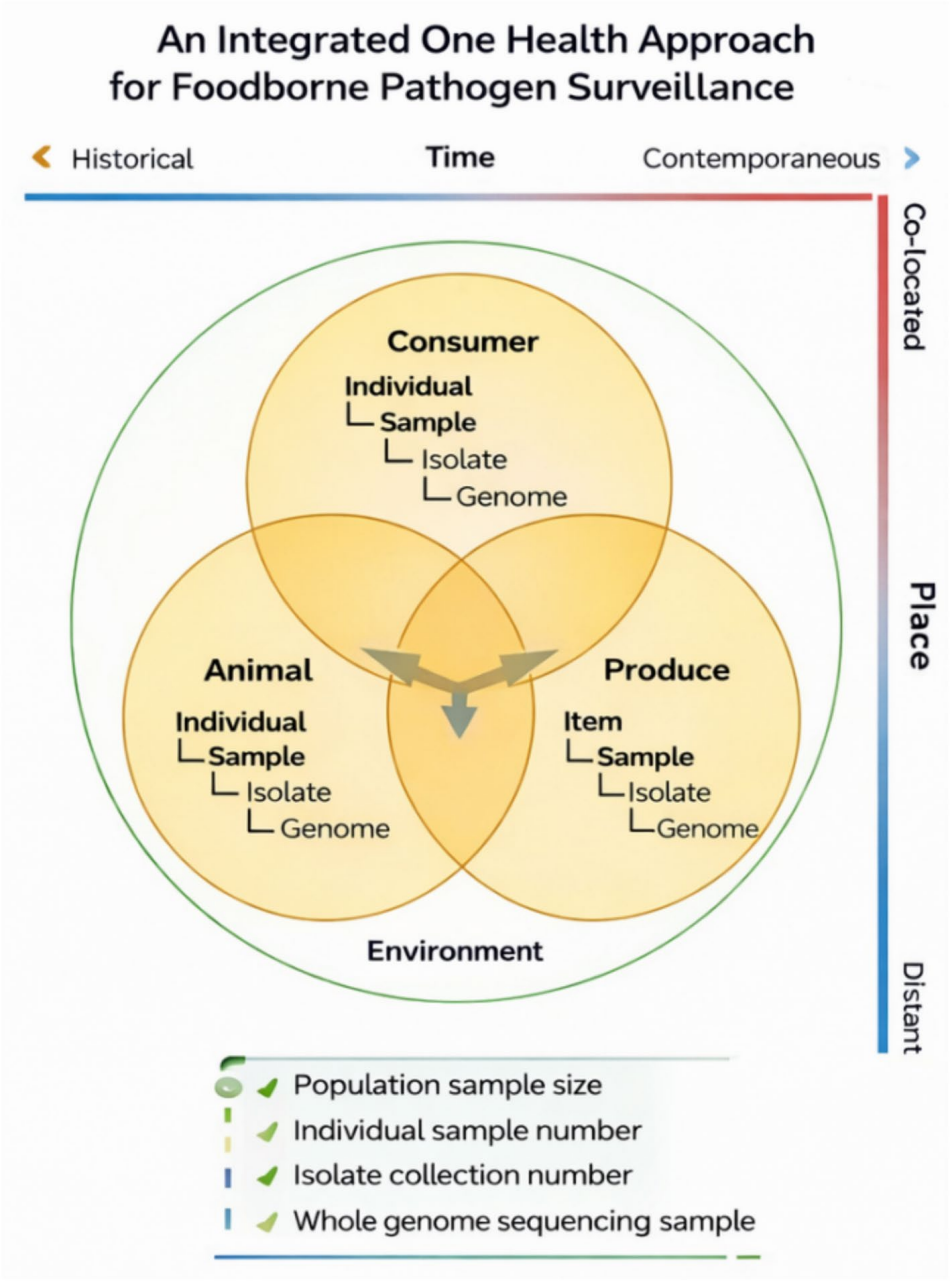
Ecological framework for integrated food safety under the One Health approach. This framework illustrates the interconnected roles of consumers, animals, and food products within the broader environmental context. It emphasizes coordinated surveillance across temporal (historical to contemporary) and spatial (co-located to distant) scales, and highlights multi-level sampling strategies ranging from population and individual sampling to isolate characterization and whole-genome sequencing.

**Figure 2:**
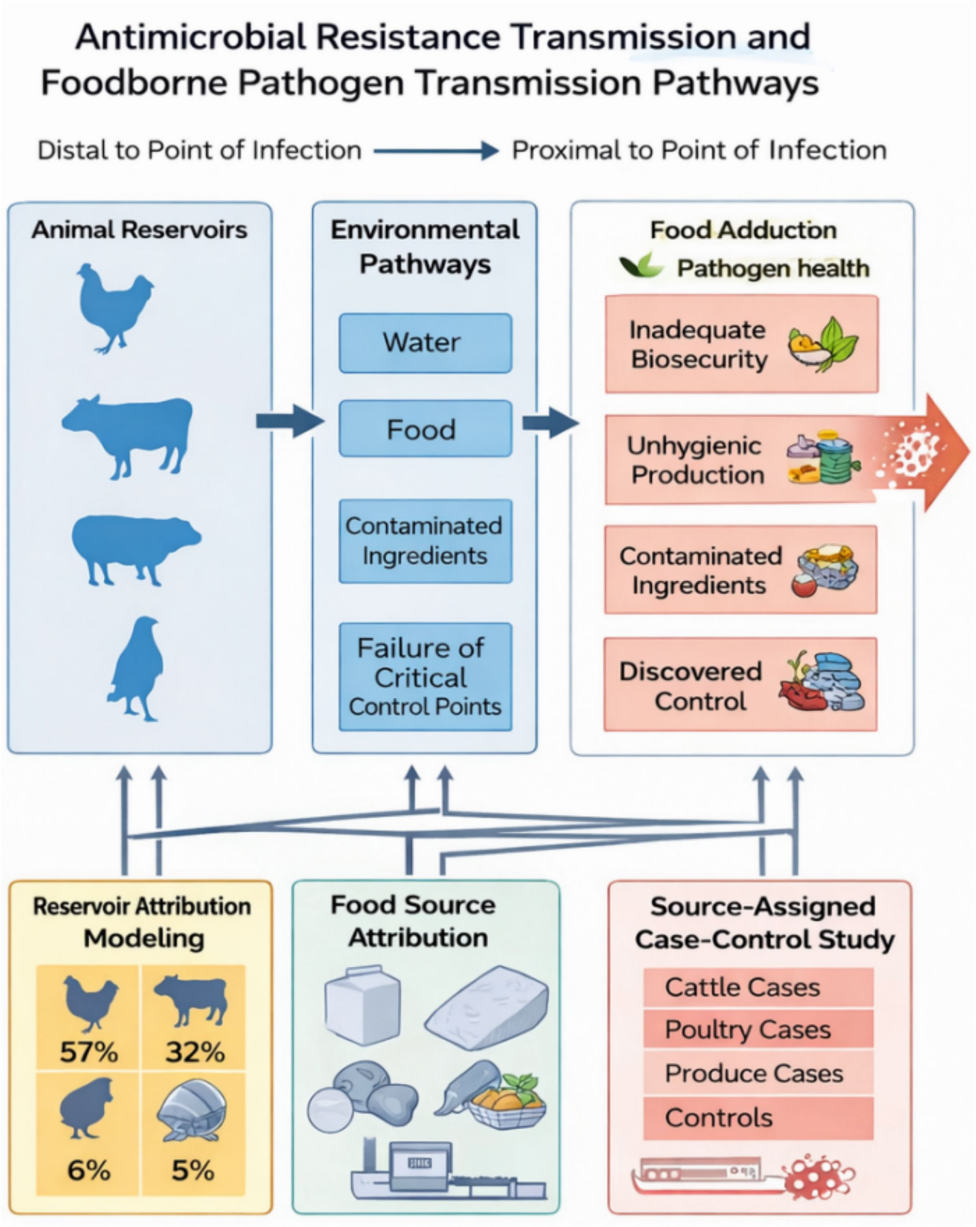
Transmission pathways of bacterial hazards along the food chain. This schematic outlines the key stages through which foodborne pathogens are transmitted from animal reservoirs to humans. Molecular approaches, including whole-genome sequencing and metagenomics, are used to identify critical transmission points and support source attribution.

To effectively prevent and control long-standing and emerging foodborne bacterial hazards, it is essential to deeply understand the influencing factors of pathogen spread in the food chain. This process spans multiple spatial and temporal scales, encompassing the dissemination of pathogens through increasingly interconnected global supply chain networks, the cross-border movement of people and animals, as well as local outbreaks or sporadic cases caused by food contamination. Many foodborne pathogens, whether long-standing or emerging, proliferate in animal hosts (reservoir hosts) before entering the food supply chain. Therefore, when monitoring bacterial foodborne hazards, it is necessary to adopt a “One Health” strategy (Ma et al., 2023). This strategy emphasizes cross-temporal and cross-spatial sampling in different environments (Figure 1) to identify the complete pathway of pathogen transmission from animal hosts to human ingestion, infection, and ultimately disease onset (Figure 2).

Genomics-based methods provide higher resolution for studying transmission processes across multiple scales, from global to local, as well as through complex food supply chains and networks. The following will elaborate on application examples of these methods, including determining historical and contemporary transmission patterns at the global scale, conducting outbreak investigations at the local and international levels, and performing source attribution and traceability tracing in the food chain.

### 4.2 Global-scale transmission

Globally, the growing food trade is a major driving force behind enhancing food security, especially for countries with rapidly growing populations and limited natural resources. However, the historical trade in live animals has spread foodborne pathogens across continents, and current trade further increases the likelihood of contaminated food moving over longer distances and at faster speeds between countries. Genome-based tools provide evidence for clarifying the role of historical trade in live cattle in the international spread of Shiga toxin-producing *Escherichia coli*, and deeply reveal the impact of transatlantic livestock trade, followed by the development of cattle farming and the industrialization of food production, on the spread of *Listeria monocytogenes*. Recently, the combination of machine learning and genome sequencing technology has enabled rapid global tracing of *Salmonella enterica*, thereby accurately locating the geographical origin of cases caused by contaminated food in the global supply chain (Bayliss et al., 2023; Franz et al., 2019; Moura et al., 2021).

## 5 Genomic epidemiology: revolutionizing surveillance and control

### 5.1 The paradigm shift enabled by whole-genome sequencing

The transition from traditional typing methods (serotyping, phage typing, PFGE) to whole-genome sequencing (WGS) represented a quantum leap in public health microbiology. WGS provided ultimate discriminatory power, turning the bacterial genome into a highly precise digital fingerprint. This capability had transformed outbreak investigation. It allowed public health officials to distinguish between sporadic, unrelated cases and those that were part of an outbreak with a common source, even when cases were scattered across wide geographical areas or over extended time periods (Allard et al., 2016; Deng et al., 2016).

As Mather et al. articulate, WGS moved food safety from a reactive to a more predictive and preventive discipline (Mather et al., 2024). In practice, this means faster and more accurate identification of the source of an outbreak, leading to more timely and targeted recalls, thereby reducing the number of illnesses. For example, during a massive, multi-year outbreak of listeriosis in South Africa linked to processed meat, WGS was critical in definitively linking clinical cases to the specific food product and processing plant (Thomas et al., 2020). In the United States, the implementation of a national WGS network (GenomeTrakr) for foodborne pathogens has demonstrably increased the number of outbreaks detected and resolved, while decreasing their size and duration (Brown et al., 2021).

### 5.2 High-resolution source attribution and risk modeling

Source attribution-quantifying the proportion of human illnesses attributable to specific animal reservoirs, food vehicles, or environmental pathways is essential for prioritizing interventions and resources. Early attribution models relied on lower-resolution typing methods like multi-locus sequence typing (MLST). WGS-based methods, including core-genome MLST (cgMLST), whole-genome single nucleotide polymorphism (wgSNP) analysis, and k-mer-based approaches, offer a dramatic increase in resolution and statistical power (Sheppard et al., 2009; Wilson et al., 2009).

These advanced models can incorporate complex datasets to provide nuanced insights. Machine learning algorithms trained on genomic data from isolates of known origin such as chicken, cattle and swine can predict the most likely source of a clinical isolate with high accuracy (Lupolova et al., 2016; Tanui et al., 2022). Furthermore, models can now integrate covariates such as geographical location, season, or patient age to reveal how attribution estimates vary under different conditions. This allows for geographically tailored intervention strategies. For instance, if a model shows that rural cases of campylobacteriosis are predominantly linked to direct contact with cattle, while urban cases are linked to retail chicken, public health messages and control efforts can be customized accordingly (Mughini-Gras et al., 2014).

Beyond traditional phylogenomic attribution models, recent studies have integrated hierarchical machine learning algorithms with genome-scale data to improve source prediction accuracy. For example, Bayliss et al. (2023) developed a machine learning framework for geographical source attribution of *Salmonella enterica* serovar Enteritidis, achieving prediction accuracies exceeding 90% using genome-derived features. By training models on well-annotated isolates of known origin, the algorithm was capable of inferring the most probable geographical source of clinical isolates within complex global supply chains.

However, despite these promising results, several challenges limit widespread implementation. First, robust model performance depends on large, high-quality, and geographically representative training datasets—conditions often unmet in low- and middle-income countries. Second, computational infrastructure and bioinformatics expertise remain unevenly distributed, creating technical barriers to adoption. Third, standardized metadata collection and harmonized data-sharing frameworks are essential for cross-border model interoperability. These constraints underscore that technological advancement alone is insufficient; equitable capacity-building and infrastructure investment are critical to realizing the full potential of machine learning enabled genomic epidemiology under the One Health framework.

### 5.3 Metagenomics and the culture-independent frontier

While WGS of isolated bacterial cultures remains the gold standard for high-resolution strain tracking, metagenomic sequencing of complex samples directly (stool, food, environmental swabs) represents a powerful complementary approach. It is culture-independent, allowing for the detection of pathogens that are fastidious, non-culturable, or present in a viable-but-non-culturable state. This agnostic approach is invaluable for discovering novel or unexpected pathogens, as demonstrated by the identification of Candidatus *Campylobacter* infans from metagenomic analysis of infant stool samples in sub-Saharan Africa and South Asia (Bian et al., 2020).

In food safety and environmental monitoring, metagenomics can be used to characterize the complete microbiome of a production facility, a food ingredient, or a watershed. Establishing a “baseline” microbiome profile allows for the detection of anomalous shifts that may indicate a hygiene failure, the introduction of a pathogen, or the proliferation of spoilage organisms (Billington et al., 2022). However, significant challenges remain. The often-overwhelming amount of host or food matrix DNA can drown out the signal from low-abundance pathogens. Determining whether a DNA sequence originates from a live, infectious organism or dead/degraded material is also difficult. Bioinformatic analysis for strain-level resolution within a complex metagenomic soup is an area of intense research (Anyansi et al., 2020; Bloomfield et al., 2023). The development of specialized bioinformatics tools, including advanced microbiome-metabolite profiling and screening platforms, has substantially enhanced our capacity to extract high-resolution information from these complex datasets, thereby generating actionable insights for public health surveillance and intervention (Yang et al., 2024; Zhang et al., 2023).

### 5.4 Data integration, sharing, and global platforms

The power of genomic epidemiology is amplified exponentially through data sharing. Global, open-access databases and platforms are crucial for tracking pathogens that know no borders. Networks like the NCBI Pathogen Detection, the Global Microbial Identifier (GMI) initiative, and platforms like Microreact and Nextstrain enable real-time comparison of sequences from across the globe (Argimón et al., 2016; Hadfield et al., 2018). During an outbreak of *Salmonella* linked to eggs from Poland, rapid international data sharing via such platforms was instrumental in recognizing the multinational scope and facilitating a coordinated response (Franz et al., 2019).

The democratization of these tools is essential. While costs have fallen, significant barriers to implementing WGS persist in low-resource settings, including infrastructure, reagent costs, and bioinformatics expertise. Global health initiatives must prioritize building sustainable capacity in these regions, not only as a matter of equity but also because pathogens emerging anywhere pose a threat everywhere. The lack of data from large parts of the world represents a critical blind spot in global health security.

## 6 Prevention and control within a one health framework

### 6.1 Integrated surveillance and antimicrobial stewardship: foundational pillars

A robust, integrated One Health surveillance system is the bedrock of effective control. This requires harmonized protocols for the collection, sequencing, and sharing of data from human, animal (livestock and wildlife), food, and environmental sectors. National and international agencies must collaborate to establish common terminologies, metadata standards, and data-sharing agreements (Baker et al., 2023; Dooley et al., 2018).

Inseparable from surveillance is the imperative for antimicrobial stewardship. Reducing the overall volume of antimicrobial use, particularly for non-therapeutic purposes in animal agriculture, is the single most effective measure to slow the development and spread of AMR. Many countries have banned the use of antibiotics as growth promoters, but therapeutic and prophylactic use remains high. Policies should promote alternative strategies for disease prevention in animals, such as improved biosecurity, vaccination, and better husbandry practices. In human medicine, guidelines must stress that uncomplicated gastroenteritis caused by NTS or *Campylobacter* typically does not require antibiotic therapy, reserving these drugs for invasive disease or high-risk patients (Wagenaar et al., 2013).

### 6.2 Interventions across the food chain: from farm to fork

A multi-hurdle approach targeting critical control points is essential. Implementing strict biosecurity to prevent pathogen introduction; using vaccines such as effective *Salmonella* vaccines for poultry; exploring novel interventions like bacteriophages, probiotics, or prebiotics to reduce pathogen colonization in animal intestines. Then, optimizing slaughter hygiene, implementing chemical or physical decontamination treatments such as organic acids, steam and ultrasound for carcasses, and employing rigorous environmental monitoring programs to control persistent strains in processing plants. Genomic data can identify “resident” strains and help validate the efficacy of sanitation protocols (Kovac et al., 2017; Mather et al., 2024). Finally, maintaining an unbroken cold chain and educating consumers on safe food handling practices are the final lines of defense. Crucial messages include preventing cross-contamination in kitchens, cooking poultry thoroughly, and avoiding consumption of raw milk or undercooked eggs.

In addition to interventions targeting animals, food processing, and consumers, environmental mitigation strategies represent a critical pillar within the One Health framework. Effective management of agricultural wastewater is essential to prevent the dissemination of foodborne pathogens and antimicrobial resistance determinants into surface water and irrigation systems. The implementation of manure composting and anaerobic digestion technologies can significantly reduce pathogen loads before land application, thereby minimizing environmental contamination and subsequent reintroduction into the food chain. Furthermore, controlling wildlife vectors and limiting their access to farms and processing environments are important measures to interrupt environmental reservoirs and cross-species transmission pathways. Integrating environmental surveillance with genomic monitoring can further support early detection of environmental persistence and improve risk assessment across interconnected human–animal–environment systems.

### 6.3 Public health measures, vaccines, and novel strategies

Public health agencies must maintain capacity for rapid outbreak response, facilitated by WGS, to quickly identify and remove contaminated products from the market. Risk communication targeted at vulnerable populations is also vital.

The development of human vaccines remains a high-priority research area, especially for endemic regions in Africa where invasive NTS disease is a major cause of childhood mortality. Genomic data aids vaccine design by identifying conserved, immunogenic targets across circulating strains. Looking forward, more fundamental, ecology-based interventions are being explored. This includes modulating the gut microbiome of food animals to make them less conducive to pathogen colonization, or using bacteriophage cocktails specifically targeted against MDR strains. Tools designed for screening microbiome metabolites linked to disease, hint at a future where we might monitor or manipulate the metabolic environment to suppress pathogenicity (Wang et al., 2023a; Yang et al., 2025a, 2025b).

Despite the advances summarized in this review, several limitations should be acknowledged. First, much of the available high-resolution genomic and surveillance data originates from high-income countries, resulting in potential geographic bias and underrepresentation of low- and middle-income regions. Surveillance systems in many resource-limited settings remain fragmented, leading to incomplete epidemiological and AMR trend assessments. In addition, global access to whole-genome sequencing and advanced bioinformatics infrastructure remains uneven, constraining equitable implementation of genomics-informed surveillance. Important knowledge gaps also persist regarding the environmental transmission dynamics of low-abundance resistance genes and the mechanistic pathways underlying post-infectious sequelae following NTS and *Campylobacter* infections. Addressing these limitations will require coordinated international investment, standardized data-sharing frameworks, and interdisciplinary research efforts.

## 7 Conclusions and future perspectives

The persistent dominance of non-typhoidal *Salmonella* and *Campylobacter* as leading foodborne pathogens is a powerful reflection of their successful adaptation to the realities of modern, globalized food production. This review has charted their significant and uneven global disease burden, the alarming acceleration of antimicrobial resistance driven by practices across the One Health spectrum, and the transformative capacity of genomic science to illuminate these complex dynamics. The evidence is clear: these are not static threats but evolving challenges shaped by biological evolution, agricultural practices, economic forces, and environmental change. The critical conclusion is that fragmented, sector-specific responses are inadequate. The path forward is unambiguously anchored in a deepened and operationalized One Health framework. This requires a dual commitment: first, to the establishment of equitable, real-time, and integrated genomic surveillance on a global scale, closing the dangerous capacity gap between high- and low-resource settings. Second, to the translation of the resulting data deluge into decisive, evidence-based action. Genomic insights must directly inform and refine national AMR stewardship policies in agriculture, guide precision food safety interventions within the industry, and enable targeted public health measures.

Future success will depend on fostering unprecedented levels of interdisciplinary collaboration and breaking down institutional silos. Economists, social scientists, ecologists, and data scientists must work alongside microbiologists, veterinarians, and public health officials. Research must expand beyond pathogen-centric views to understand the ecological networks within food systems that support pathogen persistence. It must also tackle the social and behavioral determinants of food safety from farm to household. Ultimately, controlling *Salmonella* and *Campylobacter* is more than a technical exercise in microbiology; it is a test of our ability to manage complex socio-ecological systems sustainably. By harnessing the precision of genomics within a collaborative One Health paradigm, we can move from reactive containment to proactive prevention and resilience-building. The goal is to forge food systems that not only minimize the present burden of these enduring pathogens but are also intelligently designed to anticipate and mitigate the emerging microbial challenges of the future, thereby safeguarding health, supporting economies, and promoting global food security.
